# Association between *Helicobacter pylori* Infection and Chronic Urticaria: A Meta-Analysis

**DOI:** 10.1155/2015/486974

**Published:** 2015-03-16

**Authors:** Huiyuan Gu, Lin Li, Min Gu, Guoxin Zhang

**Affiliations:** ^1^Department of Gastroenterology, First Affiliated Hospital of Nanjing Medical University, Nanjing 210029, China; ^2^Department of Pediatrics, Changzhou Children's Hospital, Changzhou, Jiangsu 213003, China

## Abstract

*Background*. Some studies have shown the possible involvement of* Helicobacter pylori* (*H. pylori*) infection in chronic urticaria, but the relationship remains controversial. The aim of this meta-analysis was to quantitatively assess the association between* H. pylori* infection and chronic urticaria.* Methods*. Observational studies comparing the prevalence of* H. pylori* infection in patients with chronic urticaria and control subjects were identified through a systematic search in MEDLINE and EMBASE up to July 2014.* H. pylori* infection was confirmed by serological or nonserological tests. For subgroup analyses, studies were separated by region, publication year, and* H. pylori* detection method to screen the potential factors resulting in heterogeneity.* Results*. 16 studies involving 965 CU cases and 1235 controls were included. Overall, the prevalence of* H. pylori* infection was higher in urticarial patients than in controls (OR = 1.66; 95% CI: 1.12–2.45; *P* = 0.01). This result persisted in subanalysis of nine high-quality studies (OR = 1.36; 95% CI: 1.03–1.80; *P* = 0.03). Subgroup analysis showed that detection method of* H. pylori* is also a potential influential factor for the overall results.* Conclusions*. Our present meta-analysis suggests that* H. pylori* infection is significantly, though weakly, associated with an increased risk of chronic urticaria.

## 1. Introduction

Chronic urticaria (CU) is a common skin disease characterized by widespread, transient wheals occurring daily or almost daily for at least 6 weeks [1]. CU may result from several causes; hidden or overt bacterial, viral, fungal, and protozoan agents have been reported as possible initiating factors, but the etiology for most cases remains unknown and therapy is largely directed at symptomatic care. Autoantibodies directed against either IgE or *α*-chain of the high-affinity IgE receptor (Fc*ε*RI*α*) can be detected in 30–50% of subjects with urticaria, suggesting that autoimmune mechanisms are involved in the pathogenesis of CU [2].

*Helicobacter pylori* (*H. pylori*) is a spiral-shaped microaerophilic Gram-negative bacterium that colonizes the gastric mucosa and induces a strong inflammatory response with release of various bacterial and host-dependent cytotoxic substances [3]. It is definitely suspected in the etiopathogenesis of gastrointestinal disorders, such as gastritis, peptic ulcer, gastric carcinoma, and lymphoma [4]. Recent epidemiological and experimental data have pointed to a strong relation of* H. pylori* infection with the development of many extragastric diseases, such as cardiovascular, immunologic, and some skin diseases [5]. Some studies have found an etiopathogenetic link between* H. pylori* infection and CU and possible skin improvement after its eradication [6–8]. Other studies, however, disagree with these findings and have found that* H. pylori* prevalence does not differ from that of control groups [9, 10]. The role of* HP* infection as a possible causative agent in CU is still controversial.

Based on current understanding of CU and* H. pylori*, we conducted this updated meta-analysis to further investigate the possible association. The existence of a correlation between* H. pylori* and CU may help clinician to find more effective methods to treat people with CU.

## 2. Methods

### 2.1. Literature Search and Selection

Two investigators (Huiyuan Gu and Lin Li) performed an initial systematic search in the databases MEDLINE and EMBASE for all relevant articles published from inception to July 2014, with the following medical subject headings (MeSH) terms and/or key words: “*Helicobacter pylori* OR* H. pylori*” AND “urticaria.” The title and abstract of studies identified in the search were reviewed to exclude any irrelevant ones. The full text of the remaining articles was examined to determine whether it contained information of interest. Moreover, we performed a manual search of references cited in the selected articles and published reviews to search for any additional relevant studies that possibly have been missed in the initial search. If required data were not provided in the article, the corresponding author of the article was contacted, whenever possible, to obtain the relevant data.

All eligible studies satisfied the following inclusion criteria: (1) they were case-control and cross-sectional study design; (2) studies provide raw data dealing with* H. pylori* infection in both CU and control groups; (3) studies include a control group with no symptoms of other skin diseases or other allergic diseases; (4) the initial diagnosis of* H. pylori* infection was made by serology, urea breath test, and histology (e.g., hematoxylin-eosin stain or Giemsa stain); at least one positive test result of these tests was considered as confirmation of infection; and (5) confirmation of CU was made according to the criteria of the European Academy of Allergy and Clinical Immunology (EAACI) or self-reported information about previous CU diagnosis provided by questionnaire or the occurrence of associated clinical symptoms.

Accordingly, studies were excluded based on the following criteria: (1) case reports, review articles, letters, editorials, comments, and observational studies without control groups; (2) studies in which the raw data of* H. pylori* infection rates were not available for either the urticaria group or the control group; (3) excluding duplicate publications or studies published only in abstract form; and (4) studies in which the participants had taken antibiotics, H_2_ blockers, proton pump inhibitors, or bismuth within the preceding 4 weeks or had serious systemic conditions.

### 2.2. Quality Assessment

The Newcastle-Ottawa scale (NOS) for case-control studies was used to assess the methodological quality of every included study. Three parameters were examined in the NOS: selection, comparability, and exposure. The highest study quality was nine stars with a maximum of four stars for selection, two stars for comparability, and three stars for exposure in the NOS. The ultimate score of 6 stars or more was regarded as high-quality [11].

### 2.3. Data Extraction

The extraction of the relevant data was performed independently by two reviewers (mentioned above). For conflicting evaluations, an agreement was reached following a discussion. For each study included, the following data were collected: first author, year, country of publication, study design, detection methods for* H. pylori* infection, and the numbers of* H. pylori* positive and* H. pylori* negative patients in the urticaria group and the control group of each study were recorded.

### 2.4. Statistical Analysis

The meta-analysis was conducted using Review Manager (RevMan, version 5.1, Copenhagen: The Nordic Cochrane Centre, The Cochrane Collaboration, 2011). Odds ratio (OR) and 95% confidence interval (CI) were estimated for each study. Statistical heterogeneity among studies was analyzed using chi-square-based *Q* statistic test and the *I* ^2^ statistic test [12]. Heterogeneity was considered statistically significant when *P* < 0.1 or *I* ^2^ > 50%. The fixed-effects model was used when there was no significant heterogeneity (*I* ^2^ < 50% and *P* > 0.1); otherwise, the random-effects model was used. The significance of the pooled ORs was determined by *Z*-test. All the reported *P* values were set two-sided, and *P* values less than 0.05 were considered to be of statistical significance. The funnel plot method was used to assess the possible presence of publication bias [13].

## 3. Results

### 3.1. Literature Search

**Figure 1:**
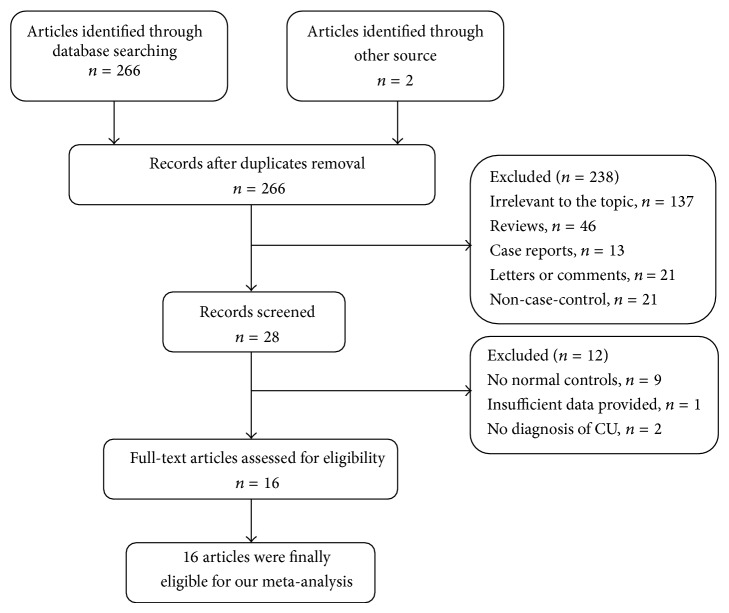
The flow diagram of the literature searches.

The detailed steps of our literature search are shown in Figure 1. A total of 268 potentially relevant articles were identified. Among these articles, 240 were excluded through review of the titles and abstracts due to duplicated publication (*n* = 2), being irrelevant to the topic (*n* = 137), not being case-control or cross-sectional studies (*n* = 21), reviews (*n* = 46), case reports (*n* = 13), or letters or comments (*n* = 21). Then, the full texts of the remaining 28 studies were carefully reviewed. Of these, 9 articles were excluded due to lack of normal control groups, one article was discarded due to insufficient data, and two articles without diagnosis of CU were also excluded. Finally, 16 studies were found to fulfill the inclusion criteria and were included in the meta-analysis [8, 9, 14–27].

### 3.2. Characteristics of Included Studies

A total of 16 studies published between 1998 and 2014 were selected for our meta-analysis [8, 9, 14–27]. The main characteristics of included studies are shown in detail in Table 1. These studies involved 2200 participants, with a total* H. pylori* infection rate of 44.73% (984/2200). The prevalence rate of* H. pylori* infection was 49.74% (480 of 965) in urticarial group and 40.81% (504 of 1235) in controls. With respect to the country of publication, there were six studies from Europe, five from Middle East, two from East Asia, one from South Asia (India), one from Southeast Asia (Indonesia), and one from South America (Brazil). According to the publication period, four studies were published from 1998 to 2000, five were published from 2001 to 2005, five were published from 2006 to 2010, and two were published from 2011 to 2015. Regarding the methods used to assess* H. pylori* infection status, ten studies used serologic methods (ELISA or western blotting or immunochromatographic method) to detect antibodies to* H. pylori*, two used urea breath test (UBT), two used histological method, and two adopted combined methods.

### 3.3. Overall Analysis

**Figure 2:**
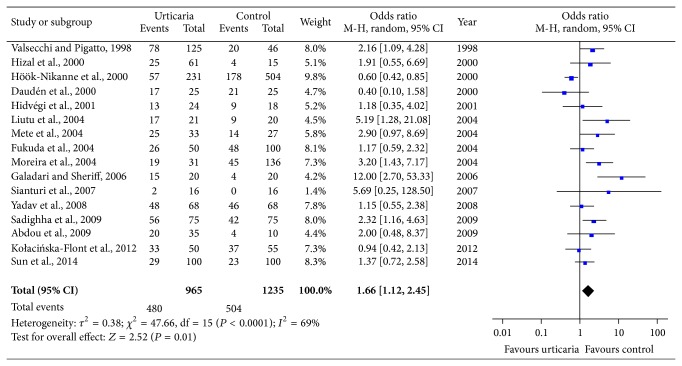
Meta-analysis for the association between* H. pylori* infection and chronic urticaria.

**Figure 3:**
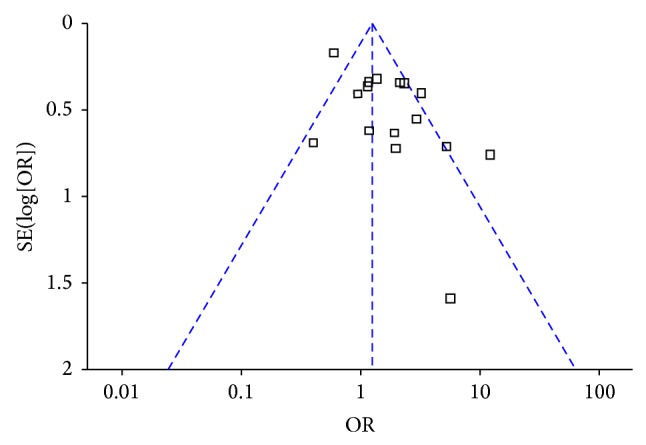
Funnel plot analysis.

Pooled data showed that the prevalence of* H. pylori* infection was higher in urticarial patients than in the controls (49.74% versus 40.81%; OR = 1.66; 95% CI: 1.12–2.45; *P* = 0.01) (Figure 2), and the test for overall effect *Z* value was 2.52, which indicated that* H. pylori* infection is associated with an increased risk of CU. Random-effect model was used for the meta-analysis due to significant heterogeneity (*I* ^2^ = 69%). In addition, the funnel plot indicated that there was no publication bias (Figure 3).

### 3.4. Subgroup Analysis

In order to explore the influencing factors that may have impacted the overall results, we carried out subgroup analyses based on region, publication period, and detection methods for* H. pylori* infection (Table 2). Stratification analysis according to geographical region indicated that the level of* H. pylori* infection was higher in urticarial patients than in the controls in the Middle East countries (OR = 2.77; 95% CI: 1.66–4.65; *P* = 0.0001). One research from South America (Brazil) showed similar result. But the difference was not significant among urticarial patients and controls in Europe, East Asia, and South and Southeast Asia. Likewise, publication year was also a potential influence factor affecting the overall result.

In this meta-analysis, various methods were adopted to confirm* H. pylori* infection. In order to exclude the probability that different methods for* H. pylori* detection would lead to different outcomes, we performed a subanalysis based on* H. pylori* detection methods. Pooled data of ten studies using serologic methods for diagnosis of* H. pylori* infection showed a weak association between* H. pylori* infection and CU (OR = 1.67; 95% CI: 1.01–2.75; *P* = 0.04). The association is prominent in two studies using combined detection methods (OR = 2.68; 95% CI: 1.27–5.65; *P* = 0.009). However, no significant associations were found in studies using UBT or histological methods as confirmation of* H. pylori* infection. Information extracted from the primary literature was insufficient to perform subgroup analyses based on age, sex, or CagA status of* H. pylori*.

### 3.5. Quality Assessment

**Figure 4:**
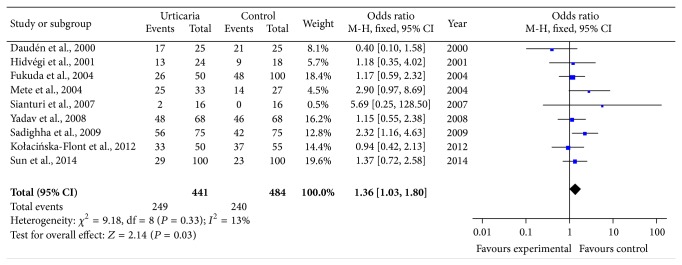
Meta-analysis of nine high-quality studies for the association of* H. pylori* infection with chronic urticaria.

The results of quality assessment according to NOS for case-control studies are shown in Table 1. Overall, the mean NOS score for all case-control studies was 6 (range: 4–7), while 9 of them were defined as high-quality [8, 16, 18, 19, 21, 24–27]. Seven studies in our meta-analysis did not adjust for confounders (such as age and sex) [9, 14, 15, 17, 18, 20, 22], which could have an effect on the study quality and our overall results. So we conducted a subanalysis involving the nine high-quality studies. As shown in Figure 4, the prevalence of* H. pylori* infection was still higher in urticarial patients than in the controls (OR = 1.36; 95% CI: 1.03–1.80; *P* = 0.03), without significant statistical heterogeneity (*I* ^2^ = 13%).

### 3.6. Sensitivity Analysis

In order to exclude the possible influence of any single research, especially low-quality research, sensitivity analysis was performed to evaluate whether omitting one study substantially altered the results or magnitude of the summary estimates of the remainders. Our sensitive analysis showed that exclusion of any study did not significantly influence the direction and magnitude of the cumulative estimates substantially (Table 3), revealing a relatively low sensitivity and high stability of our results.

## 4. Discussion

In the present study, we used meta-analytic techniques to evaluate for a possible etiological association between* H. pylori* infection and CU. Our meta-analysis of 16 studies worldwide indicated that* H. pylori* infection was higher in patients with CU than that in nonurticaria patients (*P* = 0.01), indicating the fact that* H. pylori* infection may serve as a risk factor for the development of CU. However, the summary OR and its 95% CI were just above 1 (1.66 and 1.12 to 2.45, resp.), suggesting that this positive association is weak and may have been biased by some confounders introduced by the non-high-quality studies. Thence, we conducted a subanalysis only including high-quality studies. The results still support a positive association between* H. pylori* and CU, although the pooled OR and 95% CI were both close to the borderline value 1 (OR = 1.36; 95% CI: 1.03–1.80).

The role of* H. pylori* infection in CU has long been investigated with controversial results. Several authors demonstrated that* H. pylori* eradication was associated with a remission of urticaria symptoms [9, 28–30], suggesting the possible involvement of* H. pylori* in the pathogenesis of this disorder. Abdou et al. [9] reported that the prevalence of* H. pylori* infection in chronic urticaria patients was not significantly different from that in normal control subjects, but the severity of urticarial symptoms was greater in the* H. pylori*-positive than in the* H. pylori*-negative group, and the severity of the symptoms depends on the density of bacterial infection and the intensity of the inflammatory infiltrate in the gastric biopsy, suggesting that* H. pylori* may have a role in the exacerbation of urticarial symptoms. After anti-*H. pylori* therapy, 80% CU patients positive for* H. pylori* experienced complete remission of urticarial symptoms [9]. However, Shakouri et al. [31] utilized the Grading of Recommendations Assessment, Development, and Evaluation (GRADE) system to evaluate 10 trials on the effectiveness of* H. pylori* eradication on CU and found that the benefit of* HP* eradication in patients with CU is weak and conflicting. A study in Germany showed no evidence stating that eradication of* H. pylori* improves the outcome in patients with CU [32]. Another recent study even showed that CU can be triggered by eradication of* H. pylori*, but the pathogenetic mechanisms are far from being clear [33]. However, remission or improvement in urticarial symptoms after* HP* eradication does not necessarily indicate a causal relationship between* HP* and CU, since triple therapy might eradicate other misdiagnosed subclinical infections as well [34]. Large, randomized, double-blinded, controlled trials are needed to establish the therapeutic utility of* H. pylori* eradication in patients with CU.

Several theories have been proposed to explain our finding. First, as full antigens, the bacteria themselves are able to cause allergy and immune responses. Chronic infection with* H. pylori* normally causes production of specific antibodies. For instance, IgG and IgA antibodies to 19-kDa* HP*-associated lipoprotein were found to play a role in the pathogenesis of CU [35]. When IgA-, IgG-, and IgE-mediated immune responses against* H. pylori* antigens were analyzed, some bacterial immunoresponsive proteins were identified in cases of CU [36]. Hizal et al. [15] speculate that* H. pylori* infection might trigger the production of IgE antibodies by cross-reaction between* HP* and gastric parietal cells or by causing inflammation in the gastrointestinal tract, which might facilitate the absorption of antigens. Once this occurs, the production of IgE antibodies responsible for the urticarial symptoms might continue even after the eradication of* HP*. Second, several inflammatory mediators released during the immune response to* H. pylori* infection, such as IL-1, TNF-*α*, LTC_4_ and PAF, may play a role in the pathogenesis of urticaria lesions, at least in producing a nonspecific increase in sensitivity of the cutaneous vasculature to vasopermeability-enhancing agents [37]. Third, the infection process development impairs the barrier function of the alimentary tract mucosa, thus impairing food processing. This creates conditions for allergic food particles to enter bloodstream, which is facilitated by inflammatory lesions of the intestinal tract [38]. Fourth,* H. pylori* may also upregulate the cytotoxic eosinophilic cationic protein secreted by activated eosinophils, which contributes to the etiopathogenesis of chronic urticaria [39].

Serology is the third method commonly used as a noninvasive method to diagnose* H. pylori* infection. And it is the only test which is not affected by local changes in the stomach that could lead to a low bacterial load and false-negative results of the other tests [40]. Our subgroup meta-analysis of ten researches revealed that the seropositivity of anti-*H. pylori* antibodies was higher in the urticarial patients than in control groups. We further assessed the six high-quality researches using serologic methods; the ORs were 1.48 (95% CI: 1.08–2.03, *P* = 0.02), still revealing a weak association between* H. pylori* infection and CU. However, it is important to state that serum IgG antibody detection cannot judge present infection of* H. pylori*, since serum antibodies specific to* H. pylori* would still remain for several months after successful eradication [41]. The ^13^C-UBT has high sensitivity and specificity for detecting active* H. pylori* infection [40]. And histology has been considered to be the gold standard for* H. pylori* detection [42]. In our meta-analysis, two studies used UBT to detect the presence of* H. pylori* and two used histological methods. The results showed no significant associations between CU and* H. pylori* infection in these two subgroups. However, the association is prominent in the combined detection subgroup. Currently, serology is recommended for initial screening of* H. pylori*, requiring further confirmation by histology and/or culture [42]. Therefore, two or more methods combined are better to confirm* H. pylori* infection when evaluating the correlation between these bacteria and CU.

The prevalence of* H. pylori* infection varied from region to region as well as from race to race [43, 44]. In developing countries, the prevalence of infection is as high as 90%, whereas, in developed countries, the prevalence is below 40% [45]. To minimize the influence of geographic and ethnic factors, we conducted subgroup analysis stratified by regions. We found that the association between* H. pylori* infection and chronic urticaria is prominent in the Middle East, but not in Europe, East Asia, or South and Southeast Asia. However, there was only one study included in the South America group (Brazil) and this result cannot represent the entire continent. We also assessed the two high-quality researches in the Middle East and three high-quality researches in Europe; the ORs were 2.47 (95% CI: 1.37–4.43; *P* = 0.002) and 0.84 (95% CI: 0.46–1.52; *P* = 0.56), respectively, consistent with the original analysis. More future studies, especially well designed and strictly implemented ones, are still needed to validate the association in each continent.

To explain the results better, some limitations of this meta-analysis should be acknowledged. First, some of our included studies did not adjust for confounders, which could have an effect on the study quality and overall results. Second, as the papers included in the present meta-analysis were limited to those published and listed on databases mentioned above from inception to July 2014, it is possible that some relevant published or unpublished studies, which may have otherwise fulfilled the inclusion criteria, were missed. Third,* H. pylori* infection was diagnosed by different methods and strategies among included studies, with some taking a single detection method and others using more than one method. This may have produced different positive rates on* H. pylori* infection due to advances in each technology. Finally, all studies included in our meta-analysis are with a case-control design that is more susceptible to bias than prospective cohort studies.

## 5. Conclusion

Our present meta-analysis suggests* H. pylori* infection is significantly, though weakly, associated with the risk of chronic urticaria. More clinical studies with larger sample sizes and well-designed investigations assessing confounders are required to further confirm our findings and to unearth the potential underlying mechanisms.

## Figures and Tables

**Table 1:** Characteristics of included studies.

First author	Year	Country	Region	Study design	*HP * detection method	*HP*(+) in the urticaria	*HP*(+) in the controls	Study quality		
								Selection	Comparability	Exposure
Valsecchi [14]	1998	Italy	Europe	C-C	ELISA, UBT	78/125	20/46	∗∗∗	—	∗∗
Hizal [15]	2000	Turkey	Middle East	C-C	Serum anti-*HP* IgG	25/61	4/15	∗∗	—	∗∗
Daudén [16]	2000	Spain	Europe	C-C	UBT	17/25	21/25	∗∗∗∗	∗	∗∗
Höök-Nikanne [17]	2000	Finland	Europe	C-C	Serology (ELISA)	57/231	178/504	∗∗∗	—	∗∗
Hidvégi [18]	2001	Hungary	Europe	C-C	Serology (ELISA)	13/24	9/18	∗∗∗∗	—	∗∗
Fukuda [19]	2004	Japan	East Asia	C-C	Serum anti-*HP* IgG	26/50	48/100	∗∗∗	∗∗	∗∗
Liutu [22]	2004	Finland	Europe	C-C	Serum anti-*HP* IgG, histology	17/21	9/20	∗∗	—	∗∗
Moreira [20]	2004	Brazil	South America	C-C	Serology (ELISA)	19/31	45/136	∗∗	—	∗∗∗
Mete [21]	2004	Turkey	Middle East	C-C	Serology (ELISA)	25/33	14/27	∗∗∗	∗∗	∗∗
Galadari [23]	2006	UAE	Middle East	C-C	Serology (ELISA)	15/20	4/20	∗∗	∗	∗∗
Sianturi [24]	2007	Indonesia	Southeast Asia	C-C	UBT	2/16	0/16	∗∗	∗∗	∗∗
Yadav [8]	2008	India	South Asia	C-C	Histology	48/68	46/68	∗∗	∗∗	∗∗
Abdou [9]	2009	Egypt	Middle East	C-C	Histology	20/35	4/10	∗∗∗	—	∗∗
Sadighha [25]	2009	Iran	Middle East	C-C	Serology (ELISA)	56/75	42/75	∗∗	∗∗	∗∗
Kołacińska-Flont [26]	2012	Poland	Europe	C-C	Serum anti-*HP* IgG	33/50	37/55	∗∗	∗∗	∗∗
Sun [27]	2014	China	East Asia	C-C	Serum anti-*HP* IgG	29/100	23/100	∗∗∗	∗∗	∗∗

C-C: case-control; *HP*: *Helicobacter pylori*; CU: chronic urticaria; ELISA: enzyme-linked immunosorbent assay; Ig: immunoglobulin; UBT: urea breath test.

^*^One point in Newcastle-Ottawa scale (NOS) score for quality of included studies. ^**^Two points in NOS score for quality of included studies. ^***^Three points in NOS score for quality of included studies. ^****^Four points in NOS score for quality of included studies. — stands for zero point in NOS score for quality of included studies.

**Table 2:** Subgroup analysis of the prevalence of *H. pylori* infection in CU cases versus controls.

Subgroup	Number of studies	Cases with *HP* (+)	Controls with *HP* (+)	OR (95% CI)	*P* value	Heterogeneity	
						*I* ^2^ (%)	*P*
Region							
Europe	6	215/476	274/668	1.14 [0.59–2.21]	0.70	73	0.002
Middle East	5	141/224	68/147	2.77 [1.66–4.65]	0.0001	11	0.34
East Asia	2	55/150	71/200	1.27 [0.80–2.03]	0.31	0	0.75
South and Southeast Asia	2	50/84	46/84	1.25 [0.61–2.53]	0.54	0	0.33
South America	1	19/31	45/136	3.20 [1.43–7.17]	0.005	—	—
Year							
1998–2000	4	177/442	223/590	1.01 [0.44–2.35]	0.98	78	0.004
2001–2005	5	100/159	125/301	2.13 [1.21–3.76]	0.009	39	0.16
2006–2010	5	141/214	96/189	2.48 [1.17–5.25]	0.02	52	0.08
2011–2014	2	62/150	60/155	1.19 [0.72–1.96]	0.50	0	0.48
Detection method							
Serology	10	298/675	404/1050	1.67 [1.01–2.75]	0.04	76	0.001
UBT	2	19/41	21/41	1.04 [0.08–12.83]	0.98	58	0.12
Histology	2	68/103	50/78	1.29 [0.67–2.46]	0.45	0	0.50
Combined detection	2	95/146	29/66	2.68 [1.27–5.65]	0.009	18	0.27
Total	**16**	**480/965**	**504/1235**	**1.66 [1.12–2.45]**	**0.01**	**69**	**0.0001**

*HP*: *Helicobacter pylori*; OR: odds ratio; CI: confidence interval.

**Table 3:** Sensitivity analysis after each study was excluded by turns.

Study omitted	OR (95% CI) for remainders	Heterogeneity
Valsecchi and Pigatto, 1998 [14]	1.62 [1.07, 2.47]	*I* ^2^ = 69%, *P* < 0.0001
Daudén et al., 2000 [16]	1.77 [1.19, 2.64]	*I* ^2^ = 69%, *P* < 0.0001
Hizal et al., 2000 [15]	1.65 [1.09, 2.48]	*I* ^2^ = 70%, *P* < 0.0001
Höök-Nikanne et al., 2000 [17]	1.80 [1.30, 2.49]	*I* ^2^ = 41%, *P* = 0.05
Hidvégi et al., 2001 [18]	1.69 [1.12, 2.56]	*I* ^2^ = 71%, *P* < 0.0001
Fukuda et al., 2004 [19]	1.72 [1.12, 2.64]	*I* ^2^ = 71%, *P* < 0.0001
Moreira et al., 2004 [20]	1.56 [1.05, 2.33]	*I* ^2^ = 67%, *P* = 0.0001
Mete et al., 2004 [21]	1.60 [1.07, 2.40]	*I* ^2^ = 69%, *P* < 0.0001
Liutu et al., 2004 [22]	1.56 [1.06, 2.32]	*I* ^2^ = 68%, *P* < 0.0001
Galadari and Sheriff, 2006 [23]	1.50 [1.04, 2.17]	*I* ^2^ = 64%, *P* = 0.0004
Sianturi et al., 2007 [24]	1.63 [1.10, 2.42]	*I* ^2^ = 70%, *P* < 0.0001
Yadav et al., 2008 [8]	1.72 [1.12, 2.64]	*I* ^2^ = 71%, *P* < 0.0001
Abdou et al., 2009 [9]	1.65 [1.10, 2.48]	*I* ^2^ = 70%, *P* < 0.0001
Sadighha et al., 2009 [25]	1.61 [1.07, 2.44]	*I* ^2^ = 68%, *P* < 0.0001
Kołacińska-Flont et al., 2012 [26]	1.74 [1.14, 2.65]	*I* ^2^ = 70%, *P* < 0.0001
Sun et al., 2014 [27]	1.70 [1.11, 2.62]	*I* ^2^ = 71%, *P* < 0.0001
